# How to Identify the Indications for Early Intervention in Acute Necrotizing Pancreatitis Patients: A Long-Term Follow-Up Study

**DOI:** 10.3389/fsurg.2022.842016

**Published:** 2022-04-06

**Authors:** Jiongdi Lu, Feng Cao, Zhi Zheng, Yixuan Ding, Yuanxu Qu, Wentong Mei, Yulin Guo, Yu-Lu Feng, Fei Li

**Affiliations:** ^1^Clinical Center of Acute Pancreatitis, Capital Medical University, Beijing, China; ^2^Department of General Surgery, Xuanwu Hospital, Capital Medical University, Beijing, China; ^3^Department of Pediatric, Chui Yang Liu Hospital Affiliated Tsinghua University, Beijing, China

**Keywords:** acute necrotizing pancreatitis, percutaneous drainage, infected pancreatic necrosis, persistent organ failure, complications

## Abstract

**Aim:**

To explore the indications for early intervention in patients with acute necrotizing pancreatitis (ANP) and evaluate the effect of early intervention on the prognosis of ANP patients.

**Methods:**

The clinical data of patients with ANP who underwent general surgery at Xuanwu Hospital of Capital Medical University from January 1, 2014, to December 31, 2020, were collected retrospectively. The patients were followed-up every 6 months after discharge, and the last follow-up date was June 30, 2021.

**Results:**

A total of 98 patients with ANP were included in the study. They were divided into an early group (*n*= 43) and a delayed group (*n* = 55) according to the first percutaneous drainage (PCD) intervention time (≤ 4 weeks or > 4 weeks). Body temperature, inflammatory factor levels, and the number of patients with persistent organ failure (POF) were higher in the early group than in the delayed group. After the minimally invasive intervention, the body temperature and inflammatory factors of the two groups decreased significantly, most patients with POF improved, and the number of patients with reversal of POF in the early group was higher than that in the delayed group. Although the patients in the early group required more surgical intervention than those in the delayed group, there was no significant difference in mortality, incidence of postoperative complications, total length of hospital stay, or operation cost between the two groups. During long-term follow-up, there was no significant difference in the incidence of short-term and long-term complications and overall survival between the two groups.

**Conclusions:**

Compared to patients in the delayed group, early intervention did not affect the prognosis of patients with ANP. It may be more suitable for patients with ANP with deterioration [such as POF or infected pancreatic necrosis (IPN)].

## Introduction

Acute pancreatitis (AP) is a common acute surgical condition of the abdomen. Although 80% of AP patients have mild self-limited disease, 20% of patients develop pancreatic necrosis and progress to acute necrotizing pancreatitis (ANP), and approximately one-third of patients with infected pancreatic necrosis (IPN) have significantly increased mortality (1, 2).

After the Dutch pancreatitis study group proposed and confirmed the effectiveness and safety of “step-up” minimally invasive intervention in the treatment of IPN (3), this strategy has become the preferred intervention recommended by current guidelines (4, 5). Specific measures were as follows: (1) for patients with suspected or confirmed IPN, timely antibiotic treatment should be administered in the early stage (≤4 weeks), and the intervention time should be postponed to 4 weeks after the onset of the disease, when the pancreatic necrosis is encapsulated and the boundary with the surrounding normal tissue is clear; and (2) percutaneous drainage (PCD) or endoscopic drainage (ED) of pancreatic necrotic tissue and effusion were performed to control infection. Video-assisted debridement (VAD) or endoscopic necrosectomy (EN) was performed according to the patient's condition, and laparotomy was performed when necessary.

In a recent international survey on the diagnosis and intervention time of IPN for patients diagnosed with IPN, although 55% of pancreatic experts supported antibiotic treatment first and puncture treatment after necrosis wrapping, but 45% of pancreatic experts still believe that minimally invasive intervention should be performed immediately after the diagnosis of IPN (6). Recently, a multicenter Randomized Controlled Trial (RCT) study published by the Dutch pancreatitis study group found that although early intervention did not benefit IPN patients more than delayed intervention, early intervention was an effective treatment option for IPN patients with clinical deterioration, but there was no clear indication of patients suitable for early intervention (7).

Therefore, by comparing the effects of different timing of PCD intervention on the long-term prognosis of ANP patients, this study clarified the indications for early PCD intervention in ANP patients and provided a reference for clinicians in the treatment of ANP.

## Methods

### Study Design

**Figure 1:**
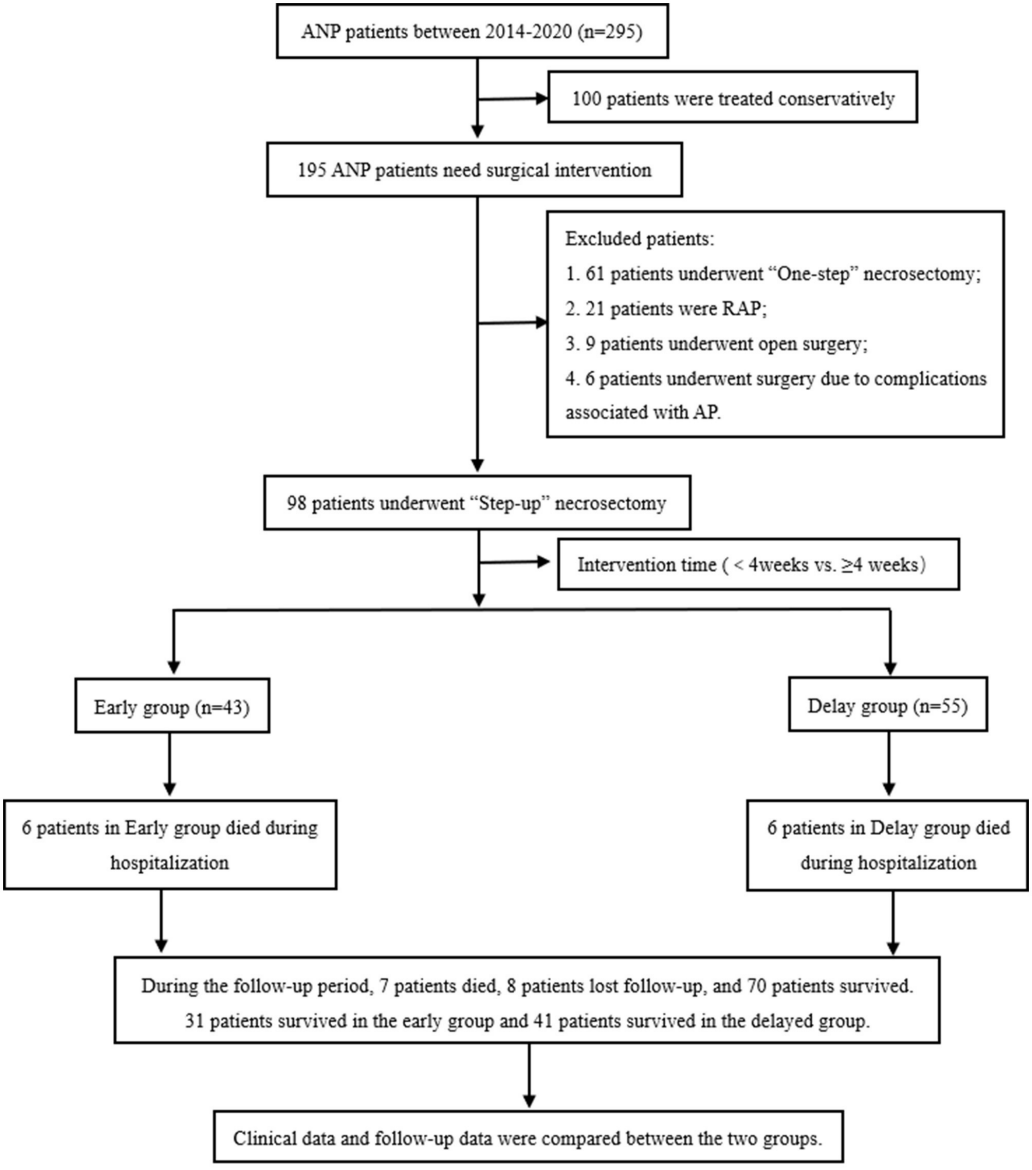
Flow chart of patient enrollment and follow-up. ANP, acute necrotizing pancreatitis; AP, acute pancreatitis; RAP, recurrent acute pancreatitis.

This study retrospectively collected the clinical data of patients with AP during general surgery at Xuanwu Hospital of Capital Medical University from January 1, 2014, to December 31, 2020, using the case database of Xuanwu Hospital of Capital Medical University. All patient data were anonymously analyzed using an electronic data acquisition system without informed consent. This study was reviewed and approved by the Ethical Review Committee of Xuanwu Hospital of the Capital Medical University (No. 2020092). A detailed flowchart of the process is shown in Figure 1.

### Inclusion and Discharge Criteria

The inclusion criteria of patients were as follows: (1) ANP patients with pancreatic and/or peripancreatic necrosis confirmed by imaging examination (enhanced CT, MRI, etc.); (2) patients were treated with the “step-up” intervention strategy; and (3) the case data and follow-up data are complete.

The exclusion criteria were as follows: (1) mild AP (MAP) without pancreatic necrosis and/or peripancreatic necrosis; (2) ANP patients with conservative treatment or “one-step” intervention strategy; (3) ANP patients requiring emergency surgery; (4) patients with chronic pancreatitis, acute attack, or recurrent acute pancreatitis (RAP); and (5) patients with incomplete case or follow-up data.

### Observation Indicators

The primary outcome of this study was number of surgical interventions and in-hospital mortality in both groups. The secondary outcomes of this study were the number of patients with persistent organ failure (POF), duration of nutritional support, type of nutritional support, operation cost, short-term postoperative complications (such as abdominal bleeding, gastrointestinal obstruction, gastrointestinal fistula, etc.), length of stay in the intensive care unit (ICU), total length of hospital stay, long-term complications during follow-up [incision hernia, pancreatic pseudocyst, RAP, pancreatic exocrine dysfunction (PEI), pancreatic endocrine dysfunction, chronic pancreatitis, pancreatic tumor, other gastrointestinal symptoms, etc.], quality of life score [Short Form-36 (SF-36), Euroqol-5 dimensions (EQ-5D) rating scales], and pain score (Izbicki pain score). The definitions of the relevant observation indicators used in this study are listed in Table 1.

**Table 1:** Definitions of the observation indicators.

**Observation indicators**	**Definition**
Acute pancreatitis (5)	Fulfillment of two of the following three criteria: (1) acute onset of epigastric pain radiating to the lower back; (2) blood amylase and/or lipase levels >3 times higher than normal; and (3) imaging examination (e.g., abdominal ultrasound, enhanced CT, and MRI) revealing typical findings of acute pancreatitis.
Necrotizing pancreatitis (8)	Presence of varying density shadows in the pancreatic parenchyma on contrast-enhanced CT, with no enhancement in the pancreatic parenchyma in the early stages of disease. The degree of pancreatic necrosis in necrotizing pancreatitis patients was divided into <30%, 30–50%, and > 50%.
Infected pancreatic necrosis (5)	Fulfillment of either of the following two criteria: (1) abdominal enhanced CT scan displaying the “bubble sign” in pancreatic and/or peripancreatic tissues; (2) development of positive pancreatic necrotic bacterial or fungal cultures with fine-needle aspiration (FNA) or other micro-invasive procedures.
**Organ failure**	
Pulmonary failure	PaO_2_/ FIO_2_ <300, or need for mechanical ventilation.
Circulatory failure	Circulatory systolic blood pressure <90 mmHg, despite adequate fluid resuscitation, or need for inotropic catecholamine support.
Renal failure	Creatinine level ≥177 umol/L after rehydration or new need for hemofiltration or hemodialysis.
New-onset organ failure	First onset of organ failure requiring intervention at any time in a 24 h period.
Multiple organ failure	Number of organs in failure ≥2.
**Surgical complications**	
Intraabdominal hemorrhage	Persistent bleeding fluid in the drainage tube or around the wound, requiring surgical, radiologic, or endoscopic intervention.
Gastrointestinal fistula	Secretion of fecal material from a percutaneous drain or inflow into the necrotic cavity, either from small or large bowel; confirmed by endoscopy, imaging or during surgery.
Gastrointestinal obstruction	Gastrointestinal symptoms (e.g., abdominal distention, abdominal pain, dyspepsia, etc.) caused by pressure on surrounding organs by pancreatic necrotic material.
Pancreatic fistula	Amylase content in drainage tube or exudate around wound ≥3 times the serum amylase level.
Abdominal compartment syndrome	An increase in intra-abdominal pressure (≥20 mmHg) caused by various factors leading to the dysfunction of digestive, circulatory, respiratory and urinary systems.
**Long-term complications**	
Incision hernia	After patient discharge, the full-thickness abdominal wall is discontinuous and abdominal contents bulge, with or without obstruction
Pancreatic pseudocyst (2)	Mature, encapsulated collection(s) of fluid with a well-defined wall outside the pancreas, homogenous fluid density, no solid component
Recurrent pancreatitis	A history of two or more episodes with and interval of at least 3 months
Pancreatic exocrine dysfunction	Clinical symptoms were improved by oral pancreatic enzyme use for more than 6 months, with no need to take this drug before the onset of AP
Pancreatic endocrine dysfunction	New onset diabetes after pancreatitis, need oral hypoglycemic drugs or insulin therapy for at least 6 months
Chronic pancreatitis (9)	Patients experience abdominal pain, weight loss, diabetes, and fatty diarrhea, endosonography/CT/MRI imaging shows dilated main duct and side branches, intraductal calcifactions, parenchymal calcifications. The symptoms did not occur before the onset of AP

### Patient Management

According to current international guidelines (5), patients were given standard treatment measures such as fluid resuscitation, analgesia, inhibition of pancreatic enzyme secretion, and early enteral nutrition after admission. Antibiotic treatment was administered only to patients with suspected or confirmed infections. Laboratory and imaging examinations were performed regularly to observe changes in the patient's condition. If the patient's condition improved, the current treatment was continued. If the patient's condition worsened, a multidisciplinary team (MDT), including pancreatic surgeons, anesthetists, intensivists, and imaging specialists, collaborated and evaluated the patients and took individualized treatment measures. Patients with deterioration organ failure (OF) or new onset OF (NOF) were provided with relevant organ support therapy [continuous pumping of vasoactive drugs, mechanical ventilation therapy (MVT), continuous renal replacement therapy (CRRT), etc.]. Patients with suspected or confirmed IPN were empirically given third-and fourth-generation cephalosporins or carbapenem antibiotics according to previous research results, which were then replaced with sensitive antibiotics according to the results of the pathogen drug sensitivity test (10).

The indications for “step-up” intervention in ANP patients were as follows: (1) after conservative treatment, the patient's condition had no significant improvement or had continued deterioration (NOF or increased temperature and inflammatory indicators, etc.); (2) the presence of IPN was confirmed; and (3) Patients develop pancreatic pseudocyst (PP) or walled-off necrosis (WON), and the range of necrosis in patients was enlarged, resulting in compression symptoms of surrounding organs (such as digestive tract or biliary tract obstruction). Pancreatic surgeons in our center have rich experience in laparoscopic necrotic tissue debridement, “PCD + VAD” was often used for intervention, and the specific intervention steps have been described in detail in previous studies (11).

**Figure 2:**
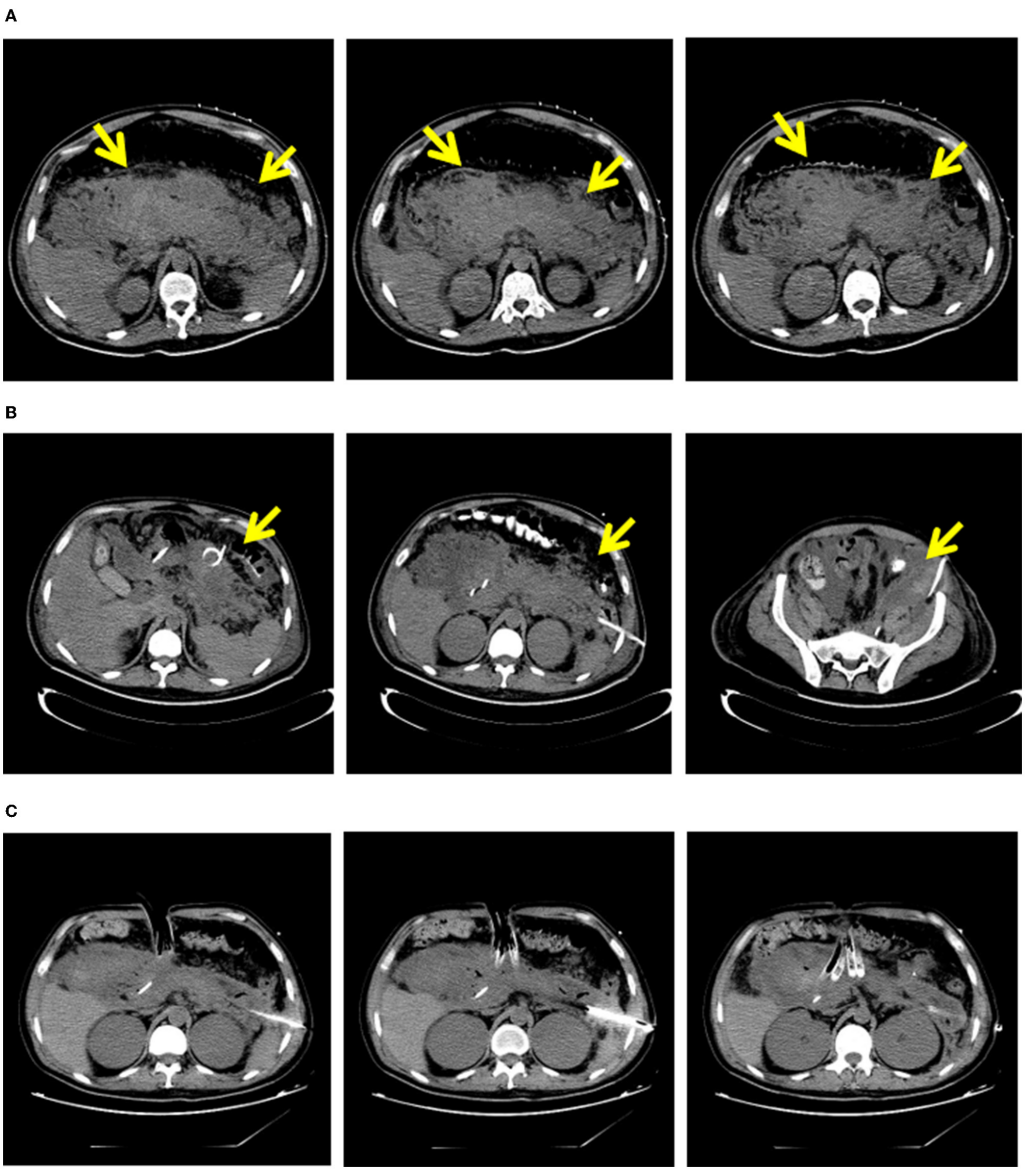
“Step-up” strategy in 32-years-old male with necrosing pancreatitis. **(A)** In 10 days of the onset of patients with pancreatic necrosis area. **(B)** Areas of pancreatic necrosis after PCD. **(C)** Areas of pancreatic necrosis after VAD. PCD, percutaneous catheter drainage; VAD, video assisted debridement.

Patients in the early group received PCD treatment within 4 weeks of onset, and patients in the delayed group received PCD treatment 4 weeks after onset. After PCD intervention, clinicians determined the next treatment strategy by observing whether the patients' clinical symptoms improved (such as reversal of OF, decrease in body temperature, decrease in inflammatory factors, and reduction of pancreatic necrosis on CT). If the patient's condition improved, current treatment was continued. If the patient's condition deteriorated, VAD treatment was performed. Representative images are shown in Figure 2.

### Follow-Up

After the patient was discharged, clinicians mainly followed-up the patients through inpatient visits, outpatient visits, telephone, e-mail, and other means. The follow-up period was 6 months. The follow-up mainly included physical examination (whether there was an incision hernia), laboratory examination (such as routine blood tests, biochemistry, and fecal elastase-1), and imaging examination (enhanced CT to evaluate whether there were morphological changes in the pancreas). In addition, patients completed the SF-36, EQ-5D, and Izbicki pain scales to facilitate the evaluation of their recent quality of life. The last follow-up date in this study was June 30, 2021.

### Statistical Analysis

In this study, Excel 2018 (Microsoft, Redmond, CA, USA) was used to record the clinical data of patients (SPSS 23.0, IBM Corp, Armonk, NY, USA), and GraphPad Prism 8.0 (GraphPad Software, La Jolla, CA, USA) was used for statistical analysis. The Shapiro–Wilk test was used to evaluate whether the study data fit the normal distribution. Data with normal distribution were expressed as mean ± standard deviation (M ± SD), and the differences between groups were analyzed using the independent sample t-test. Data with skewed distribution were presented as median (range), and between-group differences were analyzed using the rank sum test. Quantitative data are presented as rates, and differences between groups were analyzed using the chi-square test or Fisher's exact probability method. The Kaplan-Meier method was used for survival analysis. Statistical significance was set at *P* < 0.05.

## Results

A total of 98 patients with ANP were included in this study, including 69 men and 29 women, with an average age of 45.88 ± 13.95 years. There were 50 cases of biliary pancreatitis, 33 of hyperlipidemic pancreatitis, two of alcoholic pancreatitis, and 13 of other causes (eight of pancreatitis after ERCP, four of unknown cause, and one of traumatic pancreatitis). Patients with ANP were divided into an early group (*n* = 43) and a delay group (*n* = 55) according to the time from the onset of ANP to the first intervention (≤ 4 weeks or >4 weeks).

### Baseline Data

There were no significant differences between the two groups in terms of sex, age, etiology, body mass index (BMI), American Society of Anesthesiologists (ASA) score, number of combined systemic diseases, or degree of pancreatic necrosis. In terms of admission laboratory indicators, the percentage of neutrophils (83.96 ± 9.08 vs. 77.99 ± 7.81%, *P* < 0.05), C-reactive protein (CRP) (306.15 ± 213.85 vs. 175.88 ± 119.01, *P* < 0.05), procalcitonin (PCT) (1.75 ± 1.35 vs. 1.16 ± 1.01, *P* < 0.05) and interleukin-6 (IL-6) (326.36 ± 214.14 vs. 203.3 ± 173.34, *P* < 0.05) in the early group were higher than those in the delay group. In addition, the hemoglobin level in the early group was lower than that in the delay group (88.13 ± 21.79 vs. 109 ± 35.51, *P* < 0.05) (see Table 2).

**Table 2:** Baseline characteristics of acute necrotizing pancreatitis patients.

**Characteristics**	**Early group (*n* = 43)**	**Delay group (*n* = 55)**	***P*** **value**
Gender [*n* (%)]			0.31
Male	28 (65.12)	41 (74.55)	
Female	15 (34.88)	14 (25.45)	
Age [year (mean ± SD)]	44.88 ± 13.70	46.66 ± 14.36	0.538
BMI	23.92 ± 3.94	24.45 ± 4.02	0.521
Etiology [*n* (%)]			0.555
Gallstones	21 (48.84)	29 (52.73)	
Hyperlipidemia	17 (39.53)	16 (29.09)	
Alcohol abuse	0 (0)	2 (3.64)	
Others	5 (11.63)	8 (14.55)	
Systemic disease			0.376
Hypertension	14 (32.56)	8 (14.55)	
Coronary heart disease	3 (6.98)	3 (5.45)	
Diabetes	7 (16.28)	8 (14.55)	
others	23 (53.49)	31 (56.36)	
ASA [score, median (range)]	1 (1–2)	1 (1–3)	0.606
Admission temperature [°C (mean ± SD)]	37.23 ± 1.09	36.74 ± 0.57	0.048^*^
CTSI [score, median (range)]	8 (4–10)	8 (2–10)	0.495
Extent of necrosis [*n* (%)]			0.603
<30%	12 (27.91)	20 (36.36)	
30–50%	16 (37.21)	20 (36.36)	
>50%	15 (34.88)	15 (27.27)	
Degree of less-enhanced necrotic area [HU (mean ± SD)]	17.76 ± 8.29	17.11 ± 9.70	0.728
Transfer time [days (mean ± SD)]	6.09 ± 2.81	16.75 ± 12.32	0.001^*^
Transfer [*n* (%)]	33 (76.74%)	45 (81.82%)	0.374
Admission laboratory indicators [mean ± SD]		
WBC (× 109/L)	11.46 ± 6.95	10.36 ± 4.75	0.355
Percentage of neutrophils (%)	83.96 ± 9.08	77.99 ± 7.81	0.017^*^
Hb (g/L)	88.13 ± 21.79	109 ± 35.51	0.019^*^
Hct (%)	27.69 ± 6.08	30.80 ± 7.58	0.031^*^
Alb (g/L)	28.12 ± 4.34	29.83 ± 6.29	0.28
CRP (mg/L)	306.15 ± 213.85	175.88 ± 119.01	0.001^*^
PCT (ng/ml)	1.75 ± 1.35	1.16 ± 1.01	0.02^*^
IL-6 (pg/ml)	326.36 ± 214.14	203.3 ± 173.34	0.002^*^

*BMI, body mass index; ASA, American Society of Anesthesiologists; CTSI, computer tomography severity index; WBC, white blood cell count; Hb, hemoglobin; Hct, hematocrit; CRP, C-reactive protein; Alb, albumin; PCT, procalcitonin; IL-6, interleukin*.

* *P < 0.05*.

### Intervention Indication

**Figure 3:**
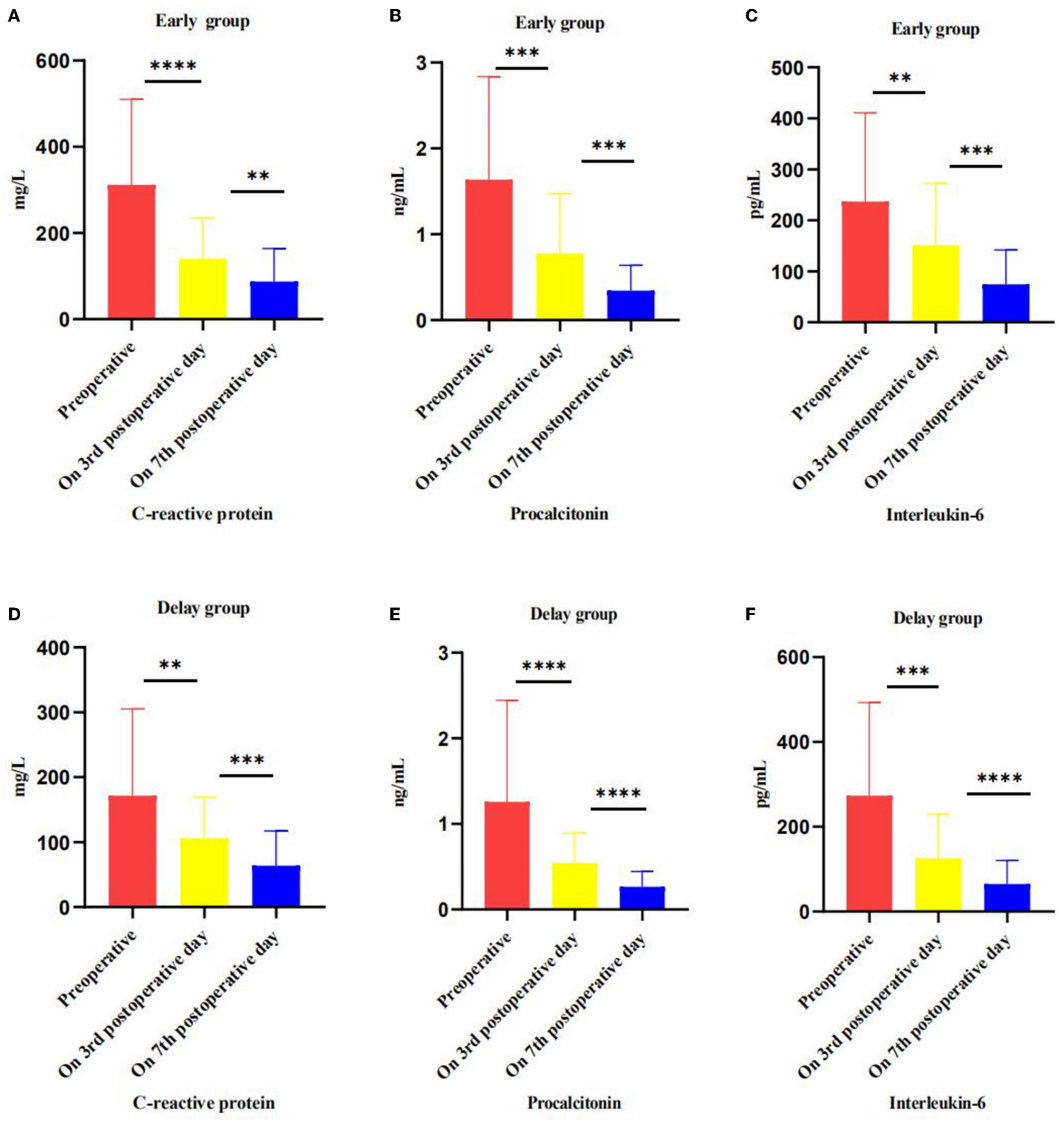
Effect of intervention on inflammatory markers, preoperative, postoperative, and pre-discharge comparison. **(A–C)** In the early group, the level of CRP, PCT and IL-6 change trend in preoperative, on 3 and 7 postoperative days. **(D–F)** In the delayed group, the level of CRP, PCT and IL-6 change trend in preoperative, on 3 and 7 postoperative days. *P < 0.05; **P < 0.01; ***P < 0.005; ****P < 0.001.

The main reason for intervention in the early group was IPN (86.05 vs. 56.36%, *P* < 0.05), while the main reasons for intervention in the delayed group were IPN and digestive tract obstruction (6.98 vs. 29.09%, *P* < 0.05). The time of first intervention in early group was earlier than that in delay group (15.26 ± 7.08 days vs. 50.86 ± 19.58 days, *P* < 0.05). he number of patients who improved after PCD treatment only (20.93 vs. 14.55%, *P* > 0.05) and the number of patients needing VAD treatment (76.74 vs. 78.18%, *P* > 0.05) were similar between the two groups. In addition, one patient in the early group and three patients in the delay group required open necrosectomy (2.33 vs. 5.45%, *P* > 0.05) (Table 3). Although the level of preoperative inflammatory factors (CRP, PCT, IL-6) in the early group was higher than that in the delayed group (*P* < 0.05), the level of inflammatory factors in both groups decreased significantly after the intervention. The results are presented in Figure 3.

**Table 3:** Intervention indications of the two groups.

**Characteristics**	**Early group** (***n*** **= 43)**	**Delay group** (***n*** **= 55)**	***P*** **-value**
Primary indications for intervention [*n* (%)]			
Infection	37 (86.05)	31 (56.36)	0.002^*^
Gastric outlet obstruction	3 (6.98)	16 (29.09)	0.009^*^
Abdominal pain	0 (0)	4 (7.27)	0.129
Other indications	3 (6.98)	4 (7.27)	0.955
Initial intervention time [days (mean ±SD)]	15.26 ± 7.08	50.86 ± 19.58	0.001^*^
Initial intervention [*n* (%)]			
PCD	9 (20.93)	8 (14.55)	0.433
Endoscopic transluminal drainage	0 (0)	1 (1.82)	0.374
Subsequent intervention [*n* (%)]			
VAD	33 (76.74)	43 (78.18)	0.805
Open necrosectomy	1 (2.33)	3 (5.45)	0.629

*PCD, percutaneous drainage; VAD, video-assisted debridement*.

* *P < 0.05*.

### Clinical Outcomes

**Figure 4:**
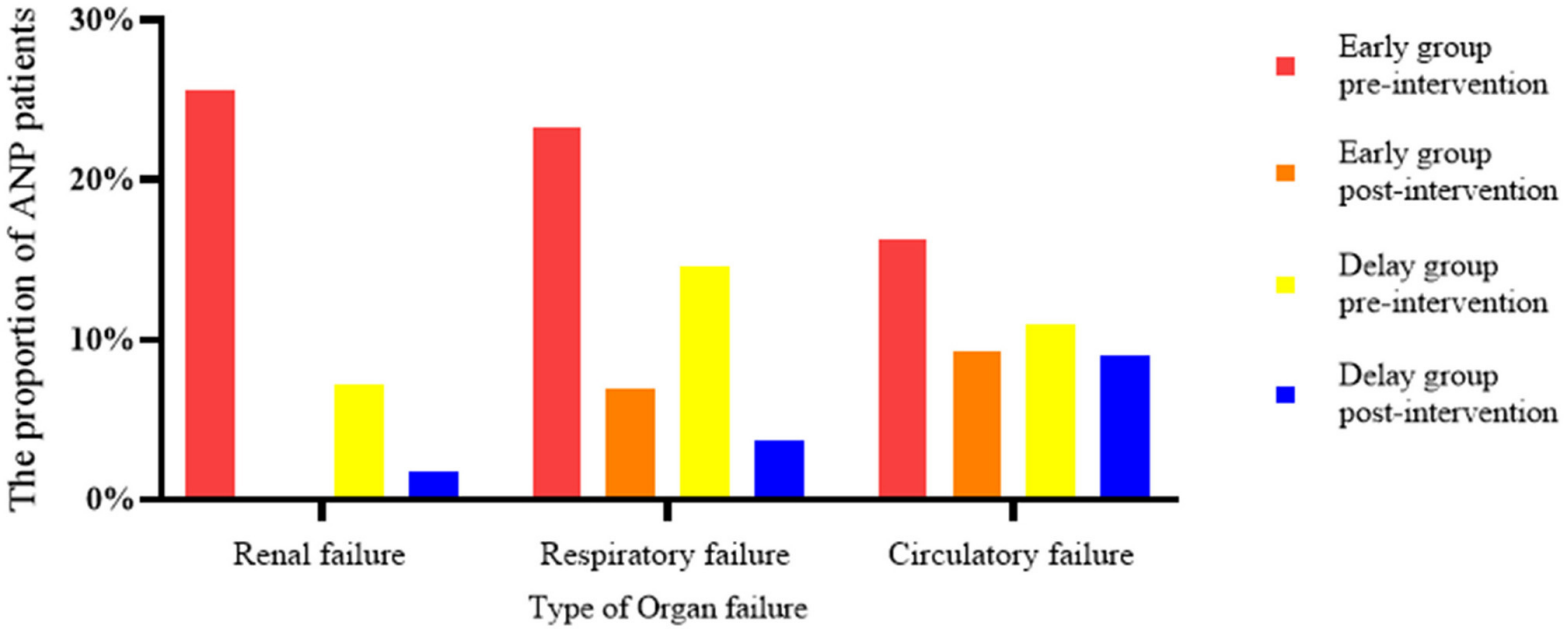
Effect of interventions on organ failure, comparing early vs. delay group. In the early group, the proportion of patients with renal failure decreased from 25.58 to 0%, that of patients with respiratory failure decreased from 23.26 to 6.98%, and that of patients with circulatory failure decreased from 16.28 to 9.30%. In the delayed group, renal failure decreased from 7.27 to 1.82%, respiratory failure decreased from 14.55 to 3.67%, and circulatory failure decreased from 10.91 to 9.09%.

In terms of clinical outcome, POF was more common in patients in the early group (44.19 vs. 18.18%, *P* < 0.05); however, most of the patients in the two groups had reversed OF after the intervention (Figure 4). Although patients in the early group needed minimally invasive intervention more than patients in the delay group [2 (1–7) vs. 2 (1–5), *P* < 0.05], the number of patients in the two groups that needed combined nutritional support (74.42 vs. 65.45%, *P* > 0.05), the duration of enteral nutritional support (22.12 ± 17.30 days vs. 26.87 ± 25.25 days, *P* > 0.05), length of parenteral nutrition support (27.54 ± 22.35 days vs. 29.61 ± 28.51 days, *P* > 0.05), operation cost (26,498 ± 9022.98 vs. 27131.92 ± 8918.18, *P* > 0.05), incidence of postoperative complications (18.60 vs. 18.18%, *P* > 0.05), length of ICU stay (25.32 ± 24.18 days vs. 30.88 ± 29.51 days, *P* > 0.05), total length of hospital stay (40.28 ± 27.52 days vs. 47.76 ± 32.51 days, *P* > 0.05), and mortality during hospitalization (13.95 vs. 10.91%, *P* > 0.05) were not significantly different (Table 4).

**Table 4:** Comparison of clinical outcomes between two groups.

**Characteristics**	**Early group (*n* = 43)**	**Standard group (*n* = 55)**	***P*** **value**
**Primary composite outcomes**			
Mortality [*n* (%)]	6 (13.95)	6 (10.91)	0.76
**Secondary outcomes**			
Persistent organ failure	19 (44.19)	10 (18.18)	0.007*
Single organ failure	10 (23.26)	2 (3.64)	
Multiple organ failure	9 (20.93)	8 (14.55)	
Renal failure	11 (25.58)	4 (7.27)	0.022*
Respiratory failure	10 (23.26)	8 (14.55)	0.302
Circulatory failure	7 (16.28)	6 (10.91)	0.552
Nutritional support [*n* (%)]			0.383
Only parenteral nutrition	11 (25.58)	19 (34.55)	
Enteral and parenteral nutrition	32 (74.42)	36 (65.45)	
Duration of nutritional support [days (mean ± SD)]			
Parenteral nutrition	27.54 ± 22.35	29.61 ± 28.51	0.685
Enteral nutrition	22.12 ± 17.30	26.87 ± 25.25	0.795
Number of operations [time median (range)]	2 (1–7)	2 (1–5)	0.03*
Surgical complications [*n* (%)]			0.794
Intraabdominal hemorrhage	2 (4.65)	4 (7.27)	
Gastrointestinal fistula	2 (4.65)	2 (3.64)	
Gastrointestinal obstruction	2 (4.65)	4 (7.27)	
Others	2 (4.65)	0 (0)	
Operation cost (RMB)	26,498 ± 9,022.98	27,131.92 ± 8,918.18	0.749
ICU stay [days (mean ± SD)]	25.32 ± 24.18	30.88 ± 29.51	0.844
Total hospital stay [days (mean ± SD)]	40.28 ± 27.52	47.76 ± 32.51	0.211

*ICU, intensive care unit*.

* *P < 0.05*.

### Follow-Up

**Figure 5:**
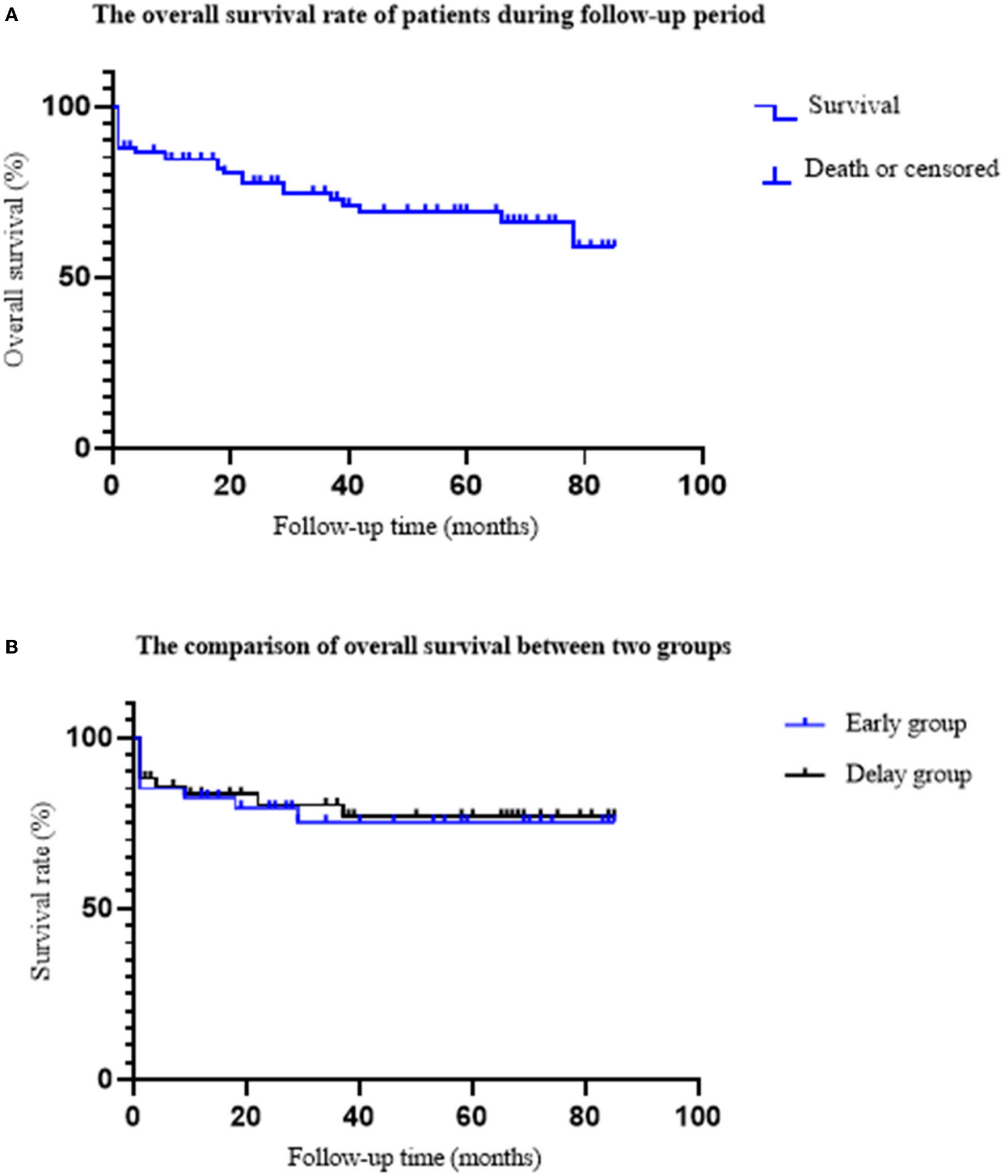
The comparison of overall survival rate between two groups. **(A)** The overall survival rate of ANP patients. A total of 19 patients died, eight patients were lost follow-up, and 71 patients survived. The average follow-up time was 40.17 ± 26.36 months. **(B)** The comparison of the overall survival rate between the two groups. In the early group, nine patients died; six patients died during hospitalization, and three patients died during follow-up. In the delayed group, 10 patients died; six patients died during hospitalization and four patients died during follow-up. The overall survival rates of early group and delayed group were 91.18 and 90.91%, respectively (*P* = 0.967).

During the follow-up period, seven patients died, eight patients were lost to follow-up, and 71 patients survived. Among them, three patients died, three patients were lost to follow-up, and 31 patients survived in the early group; in the delayed group, four patients died, five patients were lost to follow-up, and 40 patients survived. The overall survival rates of the two groups were 91.18% (31/34) and 90.91% (40/44), respectively (Figure 5).

There were no significant differences in the follow-up time (42.83 ± 25.74 vs. 41.74 ± 27.09, *P* > 0.05), pancreatic pseudocyst (8.11 vs. 4.08%, *P* > 0.05), incisional hernia (5.41 vs. 4.08%, *P* > 0.05), recurrent acute pancreatitis (24.32 vs. 8.16%, *P* > 0.05), new onset pancreatic endocrine insufficiency (29.73 vs. 14.28%, *P* > 0.05), pancreatic exocrine insufficiency (13.52 vs. 16.33%, *P* > 0.05), and chronic pancreatitis (2.70 vs. 6.12%, *P* > 0.05) (Table 5).

**Table 5:** The long-term complication between the two groups during the follow-up period.

**Characteristics**	**Early group (*n* = 31)**	**Delay group (*n* = 40)**	***P*** **value**
Follow-up time (months)	42.83 ± 25.74	41.74 ± 27.09	0.858
Long-time complications [*n* (%)]			
Pseudocyst	3 (9.68)	2 (5)	0.647
Incision hernia	2 (6.45)	2 (5)	0.792
Recurrent pancreatitis	9 (29.03)	4 (10)	0.062
New onset endocrine insufficiency [*n* (%)]			0.104
Oral medication	9 (29.03)	6 (15)	
Insulin	2 (6.45)	1 (2.5)	
Pancreatic exocrine insufficiency [*n* (%)]			0.946
Diet adjustment	1 (3.23)	0 (0)	
Enzyme use	5 (16.13)	8 (20)	
Chronic pancreatitis [*n* (%)]	1 (3.23)	3 (7.5)	0.627
Pancreatic cancer [*n* (%)]	0 (0)	0 (0)	0
Clinical symptoms [*n* (%)]			0.268
Bloating	3 (9.68)	5 (12.5)	
Weight loss	6 (19.35)	2 (5)	

In the quality-of-life rating scale, there was no statistically significant difference in the SF-36 physical or mental health score, EQ-5D health status score, or Izbicki pain score between the groups (Table 6).

**Table 6:** Quality of life rating scale during the follow-up period of surviving acute necrotizing pancreatitis patients.

**Rating scale (mean ±SD)**	**Early group (*n* = 31)**	**Standard group (*n* = 41)**	***P*** **-value**
SF-36 Physical health score^a^	40.54 ± 7.58	37.00 ± 13.89	0.18
SF-36 Mental health score^a^	45.03 ± 8.45	40.91 ± 15.24	0.157
EQ-5D based health status score^b^	71.40 ± 13.99	67.79 ± 25.99	0.463
Lzbicki pain score^c^	17.31 ± 13.85	13.47 ± 12.83	0.207

a *SF-36, Short Form-36. The SF-36 physical and mental health scores range from 0 to 100. The higher the score, the better the quality of life*.

b *EQ-5D, Euroqol-5 dimensions. The scores also range from 0 to 100, and the higher the score, the better the health*.

c *The higher the Izbicki pain score, the more severe is the discomfort. The Izbicki pain score scale includes four parts (ranging from 0 to 100 per part); the sum of the values of the four parts is divided by 4*.

## Discussion

There has always been controversy over the timing of intervention with “step-up” strategies. Some pancreatic experts supported antibiotic treatment first and puncture treatment after necrosis wrapping, these experts believe that: (1) in the early stage of the disease, the boundary between the scope of pancreatic necrosis and normal tissue is blurred. Early intervention causes great trauma to patients and is more prone to postoperative complications. (2) Some patients can improve after conservative treatment with antibiotics without intervention. Other pancreatic experts still believe that minimally invasive intervention should be performed immediately after the diagnosis of IPN (6). They considered that: (1) the concept of “delayed intervention” comes from the era of open surgery, and it is controversial whether it is applicable to the current era of minimally invasive surgery (12); and (2) in the era of minimally invasive approach, PCD is not technically difficult, and in other abdominal cases requiring PCD, drainage before necrotic wrapping has been a very common practice (13). Theoretically, timely drainage of pancreatic necrotic tissue rich in inflammatory factors is conducive to reducing systemic inflammatory response syndrome (SIRS), avoiding further clinical deterioration, and improving the prognosis of patients. In addition, PCD can reduce abdominal pressure and the risk of abdominal compartment syndrome. Furthermore, early PCD intervention can control the source of infection and speed up the encapsulated necrotic tissue (14).

By comparing the clinical data of patients in the early and delayed groups, this study defined the indications for early PCD intervention in patients with ANP. Compared with delayed intervention, early intervention did not increase mortality, incidence of postoperative complications, operation cost, or length of hospital stay. In the long-term follow-up, the overall survival and long-term complication rates of the two groups were similar. It has been further confirmed that early PCD intervention is an effective and safe treatment strategy for ANP patients with deterioration (such as POF or IPN) in the early stages of the disease.

Previous studies have pointed out that 18% of ANP patients were diagnosed with IPN (bubble sign) 3 weeks before the onset, 43% developed package necrosis 3 weeks before the onset, and have intervention indications in the early stage of the disease (15). In a large-scale multicenter study of the Dutch pancreatitis study group, the early group received PCD intervention within 24 h, and the delayed group received PCD intervention as late as 4 weeks after onset on the basis of antibiotics, and 39% of patients in the delayed group improved after conservative treatment with antibiotics, confirming the effectiveness of antibiotics in IPN patients. Although the comprehensive complication index score and mortality of the two groups were similar, it did not prove the superiority of early intervention in the treatment of patients with IPN. It is only suggested that early PCD intervention can be considered for IPN patients with rapid clinical deterioration (7). In this study, patients in the early group received PCD intervention after the failure of conservative antibiotic treatment. The first PCD intervention in most patients in the early group was between 2 and 3 weeks after onset. In addition, only 18.37% of patients with ANP showed improvement after PCD intervention. This is lower than the 35–51% reported by other research institutes (3, 7, 9, 16, 17). It may be that our hospital is one of the largest acute pancreatitis diagnosis and treatment centers in northern China. Approximately 80% of patients were referred from other hospitals, and some patients were referred to our hospital after the PCD intervention failed, and the condition was relatively serious. We believe that for ANP patients with suspected or confirmed IPN, the timing of early intervention should be determined according to the changes in the patients' condition after conservative treatment with antibiotics.

Although the current guidelines recommend that patients with ANP with suspected or confirmed infection should be treated conservatively with antibiotics, some patients still deteriorate after conservative treatment, suggesting that conservative treatment may not be applicable to all patients with IPN (5). In the expert consensus of the American Gastroenterology Association on the management of pancreatic necrosis, PCD should be considered when patients are suspected of having IPN and conservative treatment fails (18). A retrospective study based on a prospective database pointed out that in ANP patients, although early intervention is conducive to reducing the inflammatory response and improving it compared with delayed intervention, it is considered that early intervention is more suitable for ANP patients with IPN and/or POF (17). A single center RCT study of the Chinese acute pancreatitis clinical trial group found that early intervention may benefit ANP patients with POF (14), and further multicenter studies were conducted to clarify the early intervention indications of ANP patients (19). In this study, the body temperature and inflammatory factor levels of patients in the early group were significantly higher than those in the delayed group, suggesting that patients in the early group had more SIRS caused by ANP. With the extension of SIRS duration, patients are more likely to have OF and IPN, and sepsis caused by IPN may induce or aggravate OF, resulting in an increased risk of disease deterioration and death (20–22). In our study, about 44.19 and 86.05% patients in the early group have POF and IPN, respectively, which were significantly higher than those in the delayed group, also supporting this opinion. After PCD intervention, the inflammatory factor levels of ANP patients decreased significantly, and most patients with POF were successfully separated from organ support treatment. The number of patients with OF remission in the early group was greater than that in the delayed group, suggesting that early PCD intervention is conducive to controlling SIRS and reversing OF.

In terms of clinical outcomes, the overall mortality of patients in this study was 12.24%, which is similar to the mortality reported in endoscopic or surgical early intervention ANP studies in recent years (7.8–30%) (7, 14, 17). Although there was no significant difference between the two groups in terms of the length of nutritional support, incidence of postoperative complications, operation cost, length of ICU stay and mortality. But the number of surgical interventions in the early group was greater than that in the delayed group, combined with the patient's admission condition and the number of patients with POF was more than those in the delayed group, affect the rise of the number of interventions.

Through the long-term follow-up of patients with ANP after discharge, it was found that there was no significant difference in the overall survival rate and the incidence of long-term complications (incision hernia, new pancreatic endocrine insufficiency, PEI, etc.) between the two groups. This further confirms that early PCD intervention is safe and effective for patients with ANP. Previous studies have reported that IPN intervention may cause damage to adjacent pancreatic tissues, resulting in a decline in the pancreatic reserve and secretion function; 21% AP patients have RAP, and ~8% of RAP patients progress to chronic pancreatitis (CP) (23), 27% have PEI, and 37% have new pancreatic endocrine insufficiency during follow-up (24, 25). This aggravates the medical burden on patients and affects their quality of life. A study by Firkins et al. (26) confirmed that age (50–64 years old), male sex, low economic level, Elixhauser comorbidity index ≥ 3 points, components of metabolic syndrome, severe AP (SAP), and RAP are risk factors for pancreatic endocrine dysfunction, while alcoholic etiology, SAP, or ANP are high-risk factors for PEI (23, 25). Sanchez et al. (27) found that triglyceride levels were positively correlated with the risk of RAP by retrospectively collecting clinical data of patients with AP in the United States. Therefore, clinicians should strengthen the publicity and education of AP-related complications, closely monitor AP patients with high-risk factors (such as laboratory examination and imaging evaluation), and follow-up regularly to prevent and delay the occurrence of long-term complications of AP.

However, this study also has some limitations: firstly, this was a retrospective study and some of the patients were referred from other hospitals. The reason for an early or late intervention is not clear, clinical indicators were affected by the details of the clinical data at the time of referral, and there may be some statistical bias. Secondly, the first PCD intervention in some patients was performed in other hospitals. The clinical experience and operation level of pancreatic surgeons in different hospitals may also affect patient prognosis. Third, we did not compare the effect of endoscopic intervention on patients with necrotizing pancreatitis.

## Conclusions

Compared to delayed intervention, early intervention did not affect the prognosis of patients with ANP. For ANP patients with deterioration (such as POF) in the early stages of the disease, early intervention may be more suitable than conservative treatment. In view of the complex and changeable condition changes of ANP patients, further multicenter clinical trials with large sample sizes are needed to verify and identify the potential beneficiaries of early intervention.

## Data Availability Statement

The raw data supporting the conclusions of this article will be made available by the authors, without undue reservation.

## Author Contributions

FL designed and performed the research. YD and Y-LF carried out the studies and participated in collecting data. YG, WM, YQ, and Y-LF performed the statistical analysis and participated in its design. JL, FC, and ZZ wrote the manuscript. FL revised the manuscript. All authors read and approved the final manuscript.

## Funding

This study supported by Beijing Municipal Science and Technology Commission (Z171100001017077), Beijing Municipal Science and Technology Commission Clinical Diagnosis and Treatment Technology Research and Demonstration Application Project (Z191100006619038), Capital Medical Development and Research Special Project (2020-1-2012 and Z201100005520090), and Construction Project of Clinical Advanced subjects of Capital Medical University (1192070312).

## Conflict of Interest

The authors declare that the research was conducted in the absence of any commercial or financial relationships that could be construed as a potential conflict of interest.

## Publisher's Note

All claims expressed in this article are solely those of the authors and do not necessarily represent those of their affiliated organizations, or those of the publisher, the editors and the reviewers. Any product that may be evaluated in this article, or claim that may be made by its manufacturer, is not guaranteed or endorsed by the publisher.
